# Sirtuin 7 Deficiency Ameliorates Cisplatin-induced Acute Kidney Injury Through Regulation of the Inflammatory Response

**DOI:** 10.1038/s41598-018-24257-7

**Published:** 2018-04-12

**Authors:** Yoshikazu Miyasato, Tatsuya Yoshizawa, Yoshifumi Sato, Terumasa Nakagawa, Yuko Miyasato, Yutaka Kakizoe, Takashige Kuwabara, Masataka Adachi, Alessandro Ianni, Thomas Braun, Yoshihiro Komohara, Masashi Mukoyama, Kazuya Yamagata

**Affiliations:** 1 Department of Medical Biochemistry, Faculty of Life Sciences, Kumamoto University, 1-1-1 Honjo, Chuo-ku, Kumamoto, Japan; 2 Department of Nephrology, Faculty of Life Sciences, Kumamoto University, 1-1-1 Honjo, Chuo-ku, Kumamoto, Japan; 3 Department of Cell Pathology, Faculty of Life Sciences, Kumamoto University, 1-1-1 Honjo, Chuo-ku, Kumamoto, Japan; 4 Department of Cardiac Development and Remodeling, Max Planck Institute for Heart and Lung Research, Bad Nauheim, Germany

## Abstract

Cisplatin-induced acute kidney injury (AKI) has been recognized as one of cisplatin’s serious side effects, limiting its use in cancer therapy. Sirtuin 1 (SIRT1) and SIRT3 play protective roles against cisplatin-induced kidney injury. However, the role of SIRT7 in cisplatin-induced kidney injury is not yet known. In this study, we found that *Sirt7* knockout (KO) mice were resistant to cisplatin-induced AKI. Furthermore, our studies identified that loss of SIRT7 decreases the expression of tumor necrosis factor-α (TNF-α) by regulating the nuclear expression of the transcription factor nuclear factor kappa B. It has been reported that cisplatin-induced nephrotoxicity is mediated by TNF-α. Our results indicate that SIRT7 plays an important role in cisplatin-induced AKI and suggest the possibility of SIRT7 as a novel therapeutic target for cisplatin-induced nephrotoxicity.

## Introduction

Cisplatin is a highly effective anti-cancer agent used in the treatment of solid organ tumors, such as head and neck, lung, breast, testis, and ovarian cancers^1,2^. Although cisplatin has taken a leading role in cancer therapy, its use is limited by its nephrotoxicity. Cisplatin-induced acute kidney injury (AKI) has been recognized as one of its serious side effects^1–3^.

Sirtuins (SIRTs) are a family of nicotinamide adenine dinucleotide (NAD^+^)-dependent histone deacetylases that regulate a variety of biological processes, including metabolism, inflammation, DNA repair, and aging^4^. Seven SIRTs (SIRT1–7) have been identified in mammals, and these proteins display different intracellular localization, enzymatic activity, and biological functions^5^. Previous studies have shown that the kidney-specific overexpression of SIRT1 or SIRT1 activation by resveratrol ameliorates cisplatin-induced AKI^6,7^. In addition, *Sirt3*-deficient mice given cisplatin experience more severe AKI than wild-type (WT) mice^8^. These findings indicate that both SIRT1 and SIRT3 play protective roles against cisplatin-induced kidney injury. Meanwhile, SIRT7 is an NAD^+^-dependent deacetylase with high selectivity for acetylated lysine 18 of histone H3 (H3K18), which plays a role in maintaining the transformed state of cancer cells by repressing the transcription of tumor suppressor genes^9^. We reported that *Sirt7* knockout (KO) mice are resistant to high-fat diet-induced body weight gain, fatty liver, and glucose intolerance^10^. Other studies have also shown the important biological functions of SIRT7 in different tissues^11–15^. However, the role of SIRT7 in cisplatin-induced kidney injury is not known.

In this study, we investigated the role of SIRT7 in renal injury in cisplatin nephrotoxicity using *Sirt7* KO mice. We found that *Sirt7* KO mice showed protective effects against cisplatin-induced AKI. Furthermore, our studies also identified that the loss of SIRT7 decreases the expression of tumor necrosis factor-α (TNF-α), a critical proinflammatory cytokine in cisplatin-induced nephrotoxicity, by regulating the nuclear expression of the transcription factor nuclear factor kappa B (NF-κB). Our results indicate that SIRT7 plays an important role in cisplatin-induced AKI.

## Results

### Expression of SIRT7 in the Kidney

**Figure 1:**
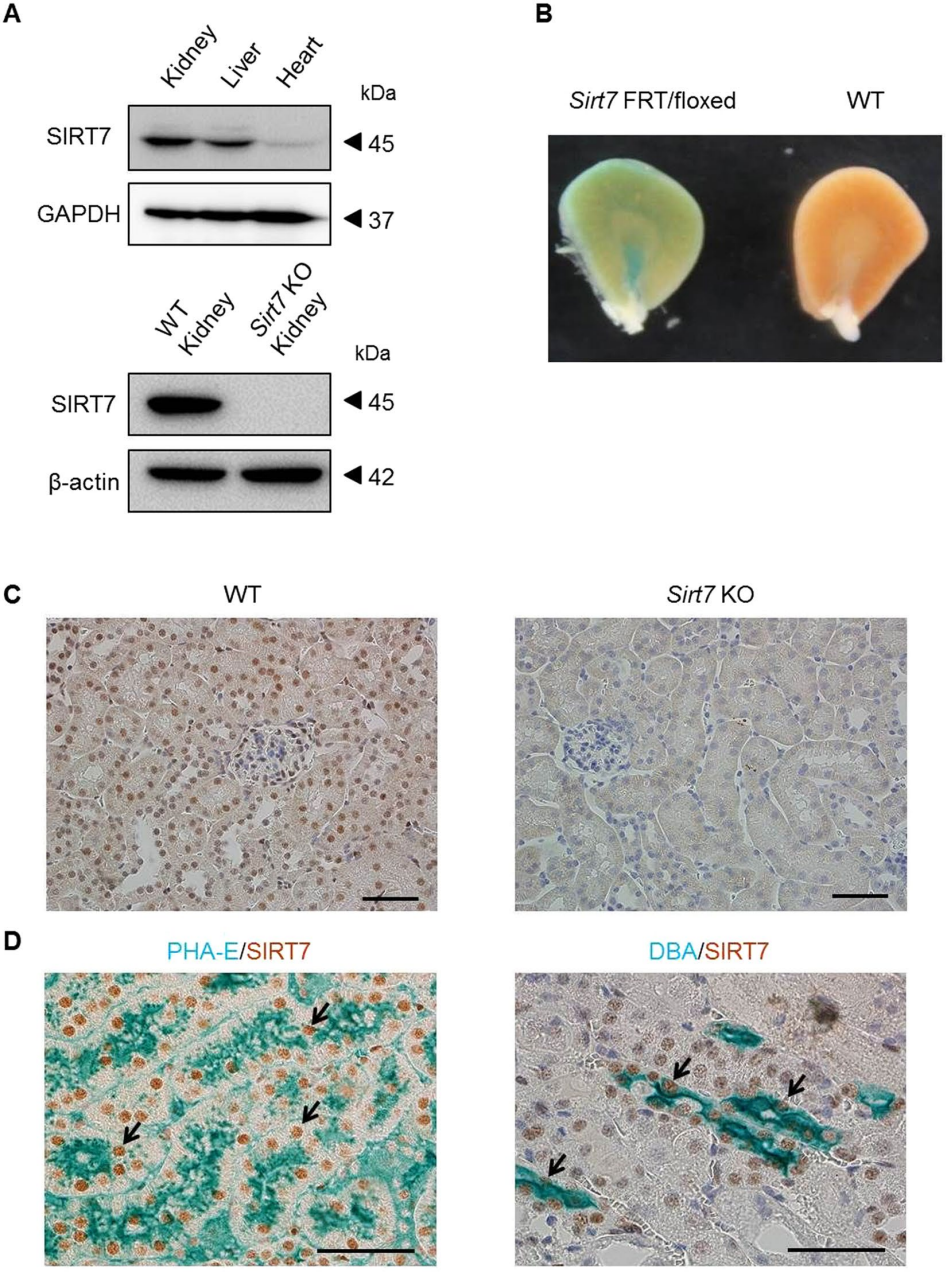
Expression of SIRT7 in the kidney. (**A**) SIRT7 expression in mouse kidney, liver, and heart was evaluated. GAPDH and β-actin were used as loading controls. SIRT7 protein expression was deleted in *Sirt7* KO mouse kidney. (**B**) SIRT7 expression in the kidney of *Sirt7* FRT/floxed mutant mouse was evaluated by β-galactosidase staining. (**C**) Representative photomicrographs (×400) of immunohistochemical staining for SIRT7 in kidney sections. Scale bar: 50 µm. (**D**) Representative photomicrographs (×400) of double immunostaining (lectin and SIRT7). PHA-E was used as a marker of proximal tubules and DBA was used as a marker of collecting tubules. The arrows indicate SIRT7-expressing nuclei. Scale bars: 50 µm.

SIRT7 is ubiquitously expressed in mouse tissues^11^. Consistent with these findings, SIRT7 protein expression was detected in the mouse kidney (Fig. 1A). The expression of *Sirt1–6* mRNA was unchanged in the kidney of *Sirt7* KO mice (Supplementary Fig. S1A). The expression pattern of SIRT7 in the kidney was assessed with *Sirt7* FRT/floxed mutant mice, which contain the *LacZ* gene under the control of the *Sirt7* promoter^10^. The inner medullary region and cortical region of the kidney were positive for X-gal staining (Fig. 1B). Parallel immunohistochemical analysis was performed using an anti-SIRT7 antibody. SIRT7 was expressed in the nuclei of renal tubular epithelial cells and glomeruli of the kidney of WT mice, but not in the kidney of *Sirt7* KO mice (Fig. 1C). Double immunostaining for SIRT7 and *Phaseolus vulgaris* erythroagglutinin (PHA-E) lectin (a marker of proximal tubular epithelial cells) revealed that SIRT7 is expressed in proximal tubular cells (Fig. 1D)^16^. Consistent with LacZ staining in the inner medullary region, SIRT7 was also expressed in *Dolichos biflorus* agglutinin (DBA; a marker of collecting duct cells)-positive collecting duct cells (Fig. 1D)^16^. SIRT7 expression in proximal tubular cells was comparable with that in collecting duct cells (Supplementary Fig. S1B,C). No overt histological abnormalities were detected in the kidney of *Sirt7* KO mice (Fig. 1C). Moreover, there were no significant differences in body weight, urine volume, and serum components between the 8-week-old WT and *Sirt7* KO mice (Supplementary Table 1).

### Cisplatin-induced Kidney Injury is Ameliorated in *Sirt7* KO Mice

**Figure 2:**
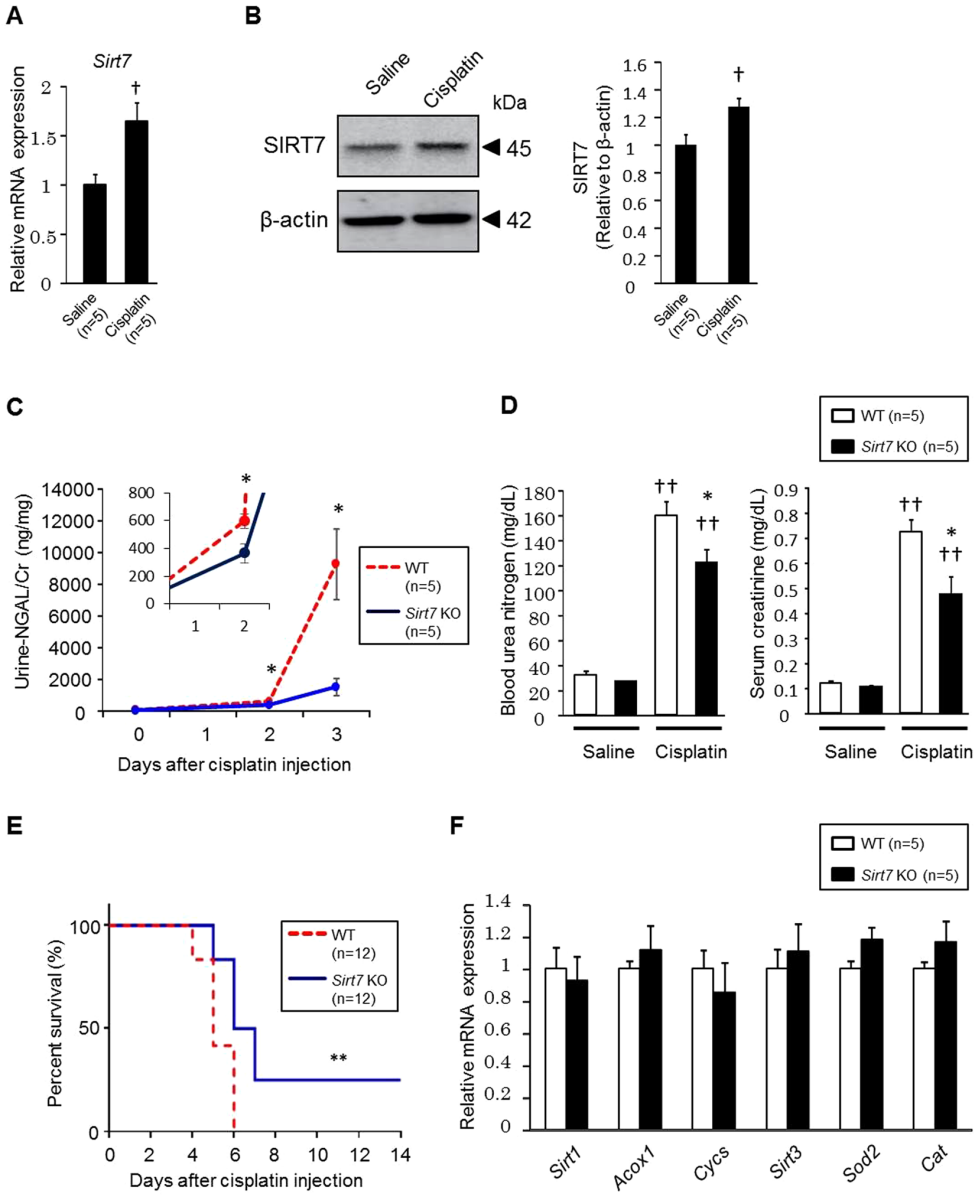
Deletion of SIRT7 ameliorates cisplatin-induced kidney injury. (**A**) *Sirt7* mRNA expression in the kidney of WT mice was determined by real-time PCR (n = 5/group). (**B**) SIRT7 protein expression in the kidney of WT mice was evaluated. β-actin was used as a loading control (n = 5/group). (**C**) Urine was collected for 24 h using metabolic cages, and urinary NGAL/creatinine levels were determined at each time point (n = 5/group). (**D**) Blood urea nitrogen and serum creatinine levels were measured at day 3 in the normal saline administration groups and cisplatin administration groups (n = 5/group). (**E**) Survival rate after cisplatin administration was assessed until day 14 (n = 12/group). (**F**) mRNA expression of *Sirt1*, *Sirt3*, and their target genes in the cisplatin-treated kidney of WT and *Sirt7* KO mice was determined by real-time PCR (n = 5/group). *WT vs. *Sirt7* KO, p < 0.05; **WT vs. *Sirt7* KO, p < 0.01; ^†^saline vs. cisplatin, p < 0.05; ^††^saline vs. cisplatin, p < 0.01. Data are expressed as the mean ± SEM.

We investigated the role of SIRT7 in cisplatin-induced AKI. Cisplatin administration led to an increase of *Sirt7* mRNA and SIRT7 protein expression in WT mice (Fig. 2A,B). We next studied the impact of SIRT7 deficiency on cisplatin-induced kidney injury. Interestingly, urinary levels of neutrophil gelatinase-associated lipocalin (NGAL), a biomarker of kidney injury^17^, were significantly lower in *Sirt7* KO mice than in WT mice (Fig. 2C). Blood urea nitrogen and serum creatinine levels were significantly lower in *Sirt7* KO mice than in WT mice at 72 h after cisplatin injection (Fig. 2D). Furthermore, *Sirt7* KO mice showed a significantly higher survival rate than WT mice after cisplatin injection (Fig. 2E). These results indicated that *Sirt7* KO mice were resistant to cisplatin-induced AKI. SIRT1 and SIRT3 play protective roles in the development of cisplatin-induced AKI^6–8^. However, the expression of *Sirt1* and *Sirt3* and their downstream target genes (SIRT1: acyl-CoA oxidase 1 [*Acox1*] and cytochrome c [*Cycs*]; SIRT3: superoxide dismutase 2 [*Sod2*] and catalase [*Cat*])^6,18,19^ was unchanged in the kidney of *Sirt7* KO mice after cisplatin administration (Fig. 2F), suggesting that the protective effect of SIRT7 deficiency is independent of these SIRTs.

**Figure 3:**
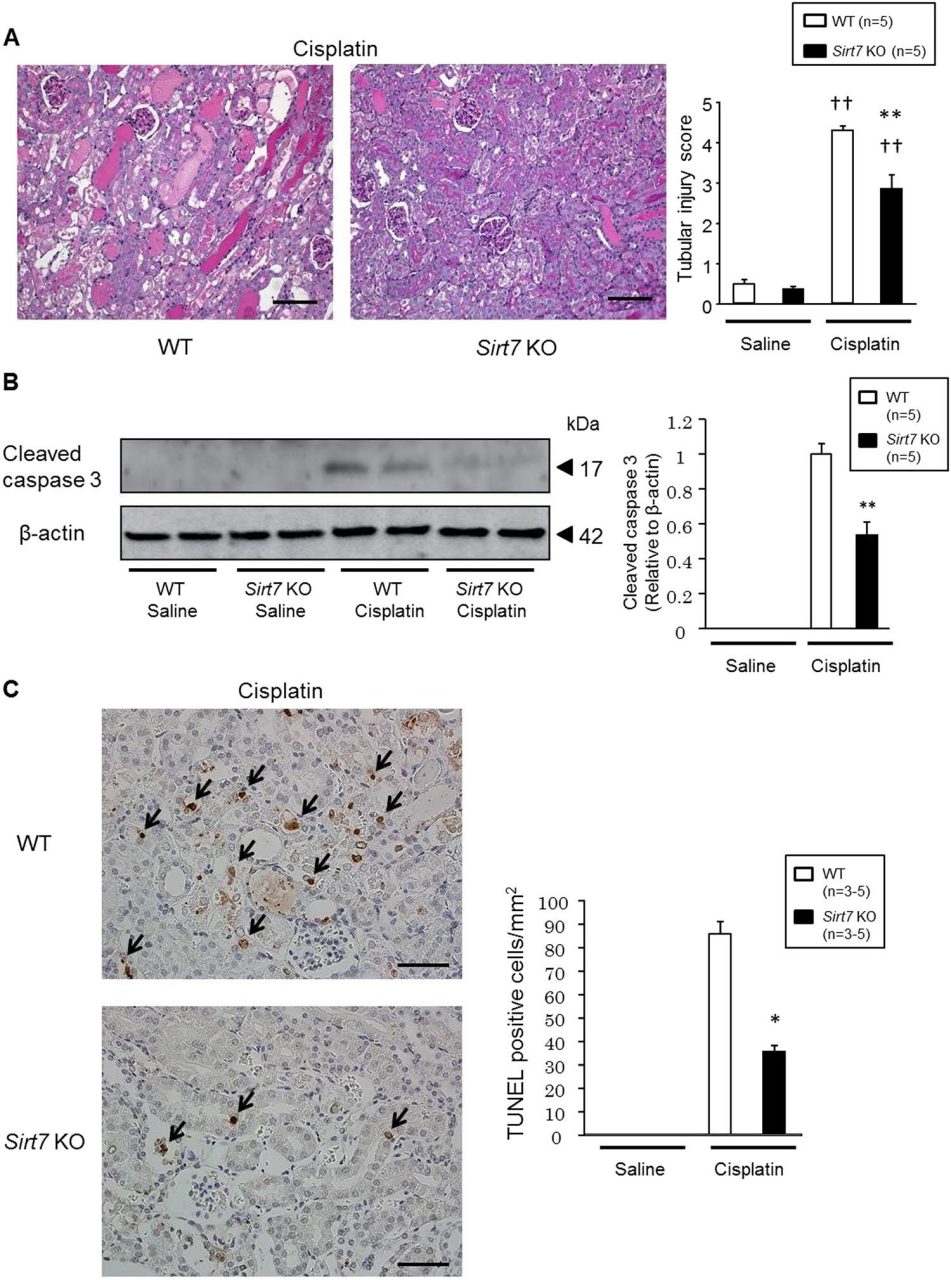
Deletion of SIRT7 ameliorates apoptosis in cisplatin-induced kidney injury. (**A**) Representative photomicrographs (×200) of PAS-stained kidney sections. The tubular injury score was calculated on a scale of 0–5 (n = 5/group). Scale bars: 100 µm. (**B**) Protein expression of cleaved caspase 3 in the kidney was evaluated by western blot analysis. β-actin was used as a loading control. (n = 5/group). (**C**) Representative photomicrographs (×400) of TUNEL staining in kidney sections. TUNEL-positive cells were counted in 4 randomly selected fields per section (n = 3/saline group, n = 5/cisplatin group). The arrows indicate TUNEL-positive cells. Scale bars: 50 µm. *WT vs. *Sirt7* KO, p < 0.05; **WT vs. *Sirt7* KO, p < 0.01; ^†^saline vs. cisplatin, p < 0.05; ^††^saline vs. cisplatin, p < 0.01. Data are expressed as the mean ± SEM.

Cisplatin mainly affects the proximal tubules in the kidney^1^. Accordingly, histological analysis revealed that cisplatin treatment resulted in severe tubular injury reflected by necrosis, cast formation, dilation, and loss of the brush border in the cortical region of WT mice (Fig. 3A). In contrast, the cisplatin-induced damage was significantly ameliorated in the cortical region of *Sirt7* KO mice (Fig. 3A). Neither WT nor *Sirt7* KO mice showed kidney tissue injury in the inner medullary region by cisplatin (Supplementary Fig. S2). Cisplatin-induced activated (cleaved) caspase 3 protein expression was detected in the kidney of WT mice, but its expression was lower in *Sirt7* KO mice (Fig. 3B). Consistent with this result, the number of TUNEL-positive cells in the injured tubules was significantly decreased in the kidney of *Sirt7* KO mice, suggesting that loss of SIRT7 suppresses the apoptosis induced by cisplatin (Fig. 3C).

### Inflammation-related Gene Expression and Macrophage Infiltration are Suppressed in the Kidney of *Sirt7* KO Mice

**Figure 4:**
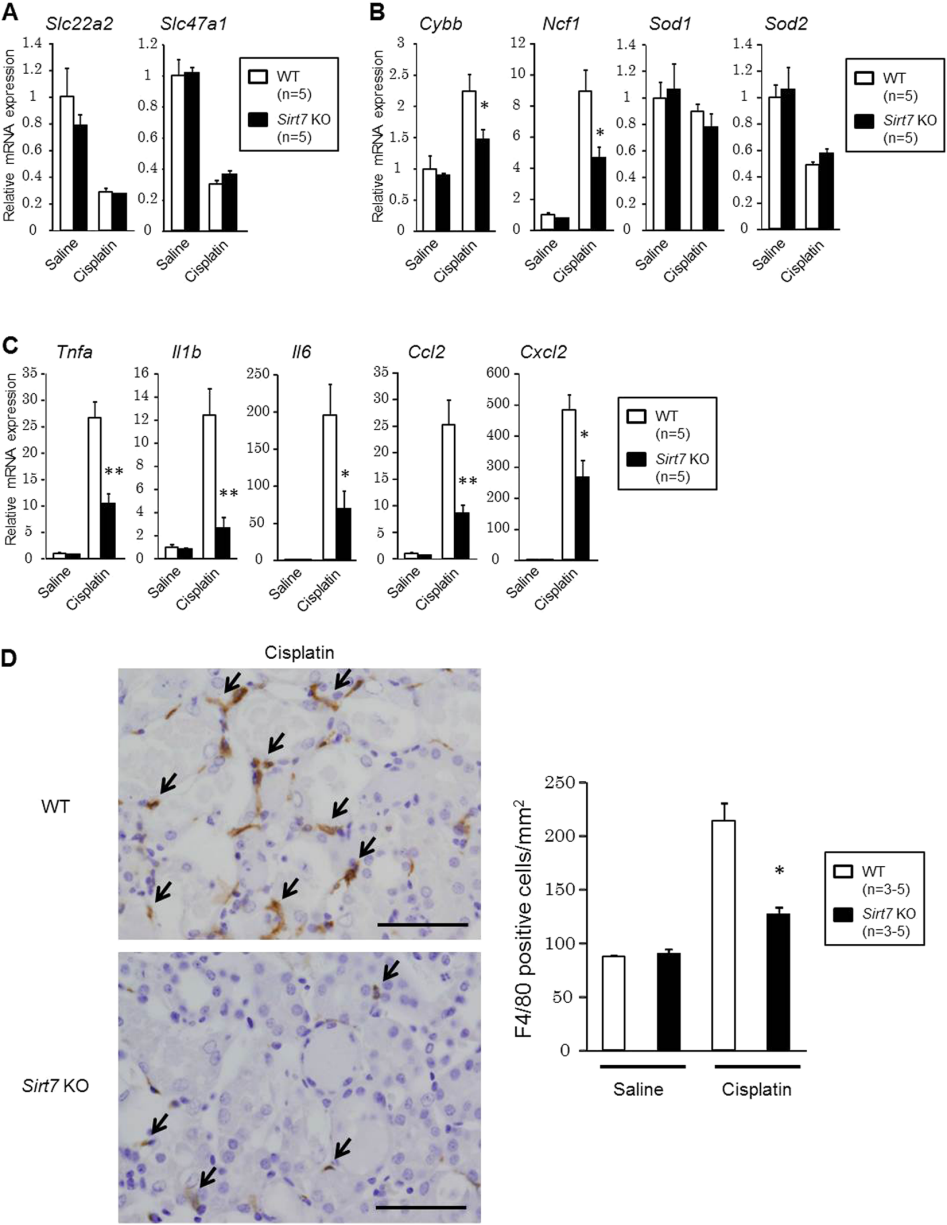
Suppression of the inflammatory response in the kidney of *Sirt7* KO mice. (**A**) *Slc22a2* and *Slc47a1* mRNA expression in mouse kidneys was determined by real-time PCR (n = 5/group). (**B**) Oxidative stress-related mRNA expression in mouse kidneys was determined by real-time PCR (n = 5/group). (**C**) Inflammation-related mRNA expression in mouse kidneys was determined by real-time PCR (n = 5/group). (**D**) Representative photomicrographs (×400) of immunostaining for F4/80 in kidney sections. F4/80-positive cells were counted in 4 randomly selected fields per section (n = 3/saline group, n = 5/cisplatin group). The arrows indicate F4/80-positive cells. Scale bars: 50 µm. The abundance of each mRNA type was normalized using GAPDH. *p < 0.05, **p < 0.01. Data are expressed as the mean ± SEM.

Next, we analyzed the expression of genes involved in cisplatin-induced renal injury. The expression of the gene encoding OCT2 (*Slc22a2*), which plays a role in cisplatin uptake^20^, and MATE1 (*Slc47a1*), which regulates the excretion of cisplatin^21^, was unchanged (Fig. 4A). Oxidative stress and inflammation play important roles in the pathophysiology of cisplatin-induced kidney injury;^1–3^ therefore, we evaluated oxidative stress and inflammation-related gene expression. The expression of the genes encoding NOX2 (*Cybb*) and p47phox (*Ncf1*), which are involved in the production of reactive oxygen species (ROS), was significantly decreased in the cisplatin-treated kidney of *Sirt7* KO mice (Fig. 4B). The expression of genes involved in inflammation (*Tnfa*, *Il1b*, *Il6*, *Ccl2*, and *Cxcl2*) was also significantly reduced in the cisplatin-treated kidney of *Sirt7* KO mice (Fig. 4C), suggesting that SIRT7 plays critical roles in the regulation of oxidative stress and inflammation.

Unilateral ureteral obstruction (UUO) is another experimental model of renal injury^22^. Consistent with the cisplatin model, the expression levels of *Tnfa*, *Il1b*, *Il6*, *Ccl2*, and *Cxcl2* at an acute stage were significantly decreased in the obstructed kidney of *Sirt7* KO mice (Supplementary Fig. S3).

In cisplatin nephropathy, the production of cytokines and chemokines by renal parenchymal cells leads to the recruitment of inflammatory cells^1^. In accordance with the reduced expression of *Ccl2* (encoding MCP1) (Fig. 4C), immunostaining for MCP1 showed a decrease of intensity in the cisplatin-treated kidney of *Sirt7* KO mice (Supplementary Fig. S4). The number of F4/80-positive infiltrating macrophages in the interstitium was significantly suppressed in the kidney of *Sirt7* KO mice as compared with that in WT mice (Fig. 4D).

### SIRT7 Regulates the Expression of TNF-α

**Figure 5:**
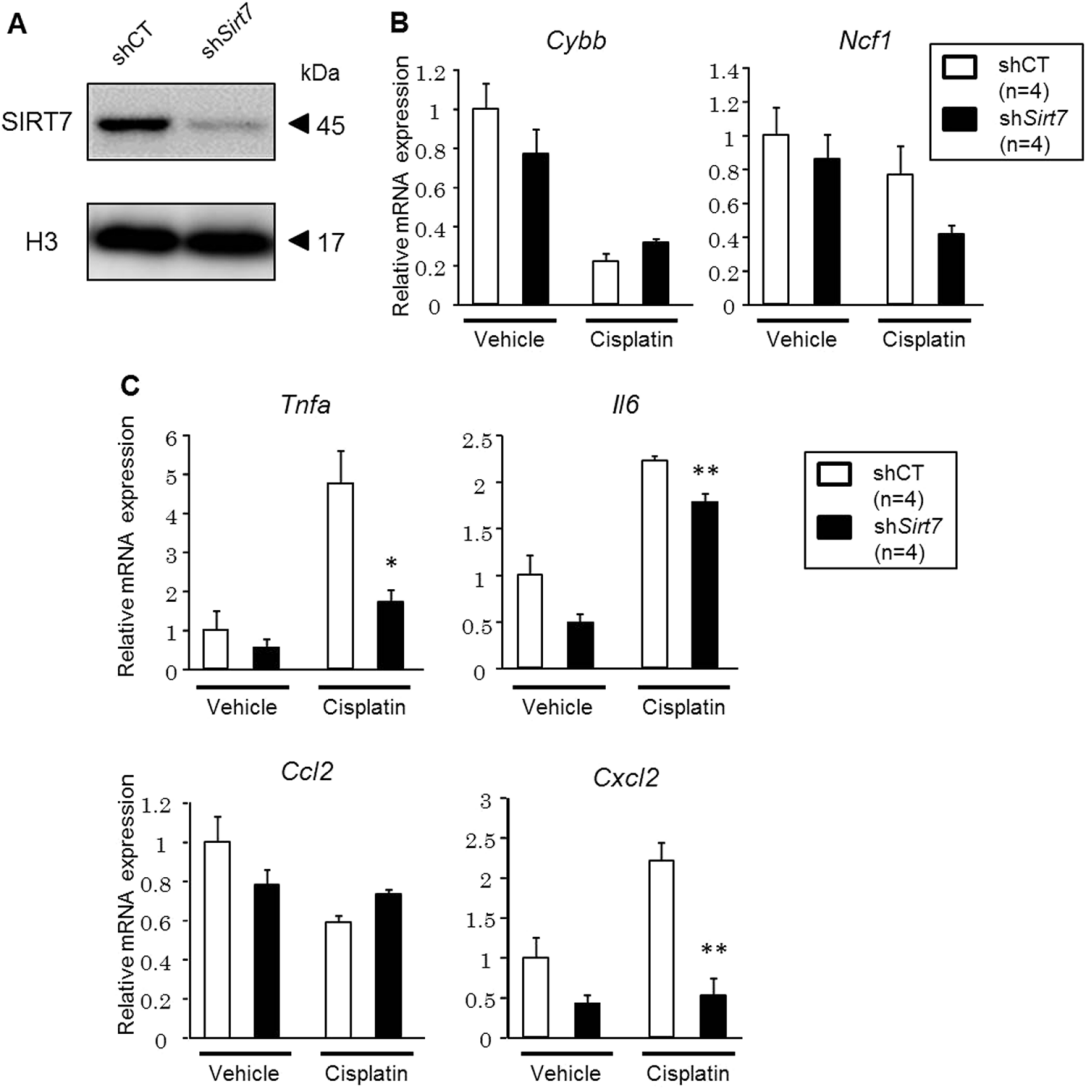
Knockdown of *Sirt7* decreases the expression of TNF-α in cisplatin-treated NRK-52E cells. (**A**) SIRT7 protein expression in shRNA-introduced NRK-52E cells was evaluated by western blot analysis. (**B**) Oxidative stress-related mRNA expression in shRNA-introduced NRK-52E cells was determined by real-time PCR (n = 4/group). (**C**) Inflammation-related mRNA expression in shRNA-introduced NRK-52E cells was determined by real-time PCR (n = 4/group). The abundance of each mRNA type was normalized using GAPDH. *p < 0.05, **p < 0.01. Data are expressed as the mean ± SEM.

We next examined gene expression in control and *Sirt7* knockdown (KD) rat renal proximal tubular NRK-52E cells. The introduction of *Sirt7* short hairpin RNA (shRNA) markedly reduced the levels of endogenous SIRT7 (Fig. 5A). The expression of *Cybb*, *Ncf1*, and *Ccl2* mRNA was reduced in the cisplatin-treated kidney of *Sirt7* KO mice (Fig. 4B,C), but their expression was not decreased in *Sirt7* KD NRK-52E cells after cisplatin exposure (Fig. 5B,C). In contrast, the expression of *Tnfa*, *Il6*, and *Cxcl2* (encoding MIP-2α) mRNA was significantly decreased in *Sirt7* KD NRK-52E cells following cisplatin administration (Fig. 5C). The expression of *Il1b* mRNA in the cisplatin-treated cells was below the detection limit. These findings suggest that SIRT7 regulates the cisplatin-induced gene expression of *Tnfa*, *Il6*, and *Cxcl2* via a cell-autonomous mechanism. Cisplatin treatment significantly induced TNF-α protein production in control NRK-52E cells, but resulted in no increase of TNF-α protein production in *Sirt7* KD NRK-52E cells (Supplementary Fig. S5). TNF-α plays a critical role in renal injury and the increase of MIP-2α expression after cisplatin administration^23,24^, whereas it has been reported that IL-6 does not play an injurious role in cisplatin-induced AKI^25^. These results suggest that SIRT7 controls cisplatin-induced nephrotoxicity, at least partly by regulating the expression of TNF-α.

### SIRT7 Regulates the Inflammatory Response Through Modulating NF-κB p65 Transcription Activity and Nuclear Translocation

**Figure 6:**
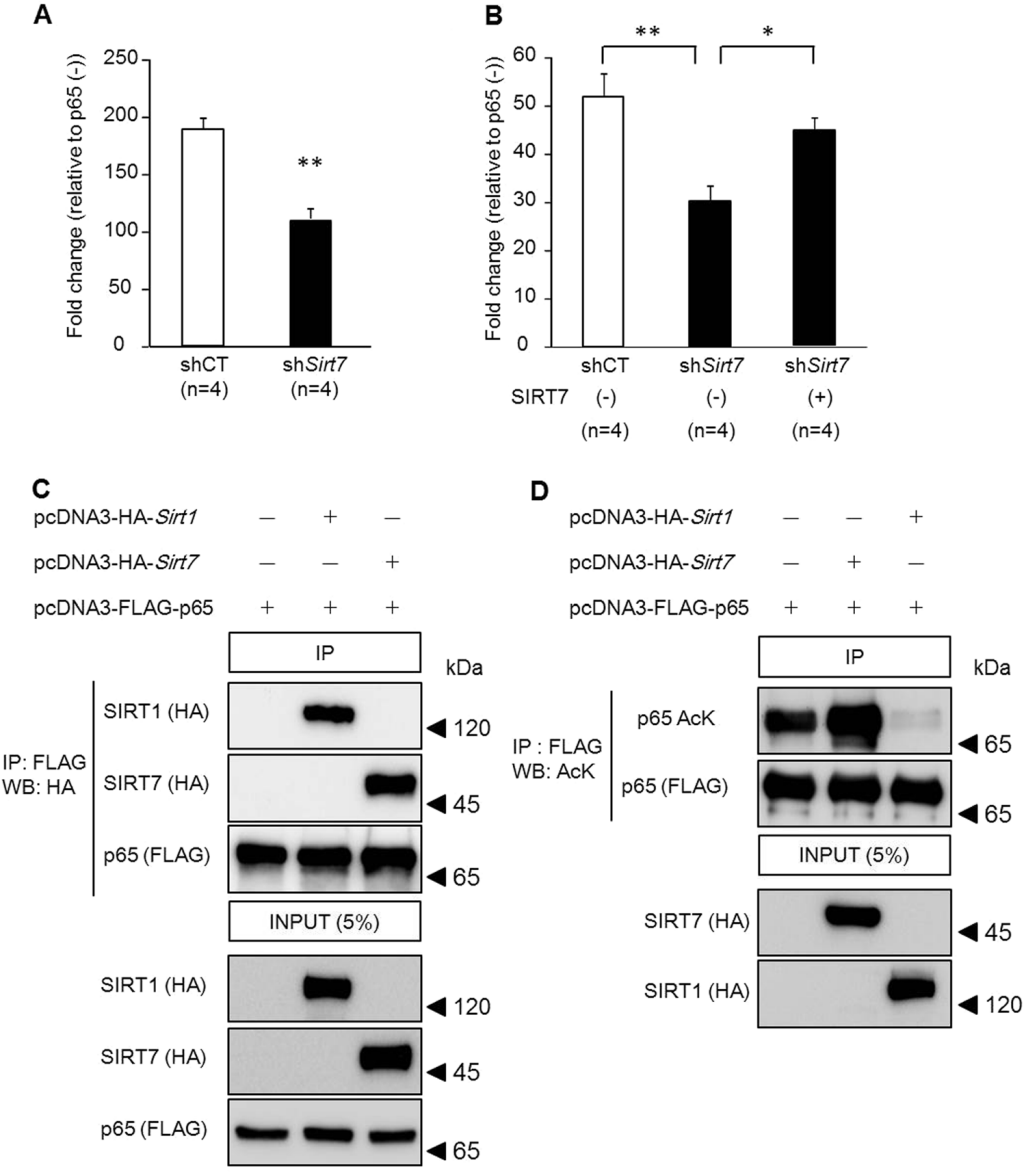
Regulation of the transactivation activity of NF-κB by SIRT7. (**A**,**B**) The transcriptional activity of NF-κB was examined using the dual luciferase reporter assay. Each *Sirt7* KD (sh*Sirt7*) and control (shCT) NRK-52E cell was transfected with 80 ng pCI-HA-p65 expression plasmid or pCI-HA-control plasmid as well as 20 ng pNF-κB-Luc plasmid, which contains multiple copies of the NF-κB consensus sequence, and 0.8 ng pRL-TK plasmid (n = 4/group). In addition, *Sirt7* KD cells were co-transfected with 100 ng pcDNA3-FLAG-*Sirt7* expression plasmid or pcDNA3-FLAG-control plasmid (**B**) (n = 4/group). (**C**) HEK293T cells were transfected with 2 μg pcDNA3-HA-*Sirt1* expression plasmid or pcDNA3-HA-*Sirt7* expression plasmid as well as 2 μg pcDNA3-FLAG-p65 expression plasmid. At 24 h after transfection, an immunoprecipitation assay and western blotting were performed. (**D**) HEK293T cells were transfected with 1 μg pcDNA3-HA-*Sirt1* expression plasmid or pcDNA3-HA-*Sirt7* expression plasmid as well as 0.5 μg pcDNA3-FLAG-p65 expression plasmid and 0.5 μg pCMVβ-*p300*-myc expression plasmid. After 36 h, an immunoprecipitation assay was performed and p65 acetylation was detected by western blotting. *p < 0.05, **p < 0.01. Data are expressed as the mean ± SEM.

The NF-κB transcription factor, composed of a heterodimer of p50 and p65 subunits, is a key mediator of the inflammatory response, including TNF-α, IL-6, and MIP-2α production^26–29^. In non-stimulated cells, NF-κB largely resides in the cytoplasm where it is bound by its inhibitory IκB family proteins. After stimulation, IκB proteins are phosphorylated by IκB kinase and degraded by the ubiquitin proteasome system^30,31^. Degradation of IκB proteins liberates NF-κB, allowing it to translocate to the nucleus. SIRT1, 2, and 6 regulate the function of NF-κB by interacting with p65^32–34^. Thus, we examined the effect of SIRT7 on NF-κB activity. Control and *Sirt7* KD NRK-52E cells were transfected with a p65 expression plasmid together with a pNF-κB luciferase reporter gene. Transfection of p65 activated the reporter gene by 188.9-fold in control NRK-52E cells. However, the transactivation activity of p65 in *Sirt7* KD cells was significantly decreased by 41.6% (p < 0.01) (Fig. 6A). Overexpression of SIRT7 in *Sirt7* KD NRK-52E cells restored the transactivation of NF-κB activity (Fig. 6B). These results indicate that NF-κB activity is reduced in SIRT7-deficient NRK-52E cells. Previous studies have revealed that FAF1, NME1, SNIP1, ING4, NFκBIL1, and TNFAIP1 regulate the activity of NF-κB in various ways^35–41^. Since SIRT7 is reported to bind to the proximal promoter regions of these genes^9^, SIRT7 might increase NF-κB activity by affecting their gene expression. However, the expression levels of these genes were similar between control and *Sirt7* KD NRK-52E cells (Supplementary Fig. S6).

SIRT1 regulates the activity of NF-κB through the deacetylation of p65. We then examined whether SIRT7 can also deacetylate p65. A co-immunoprecipitation assay revealed that SIRT7 as well as SIRT1 bound to p65 in HEK293T cells (Fig. 6C). Deacetylation of p65 by SIRT1 was detected in these cells (Fig. 6D, lane 3) as described previously^32^, while SIRT7 overexpression did not decrease the acetylation status of p65 (Fig. 6D, lane 2). These results indicate that SIRT7 has no deacetylase activity for p65.

**Figure 7:**
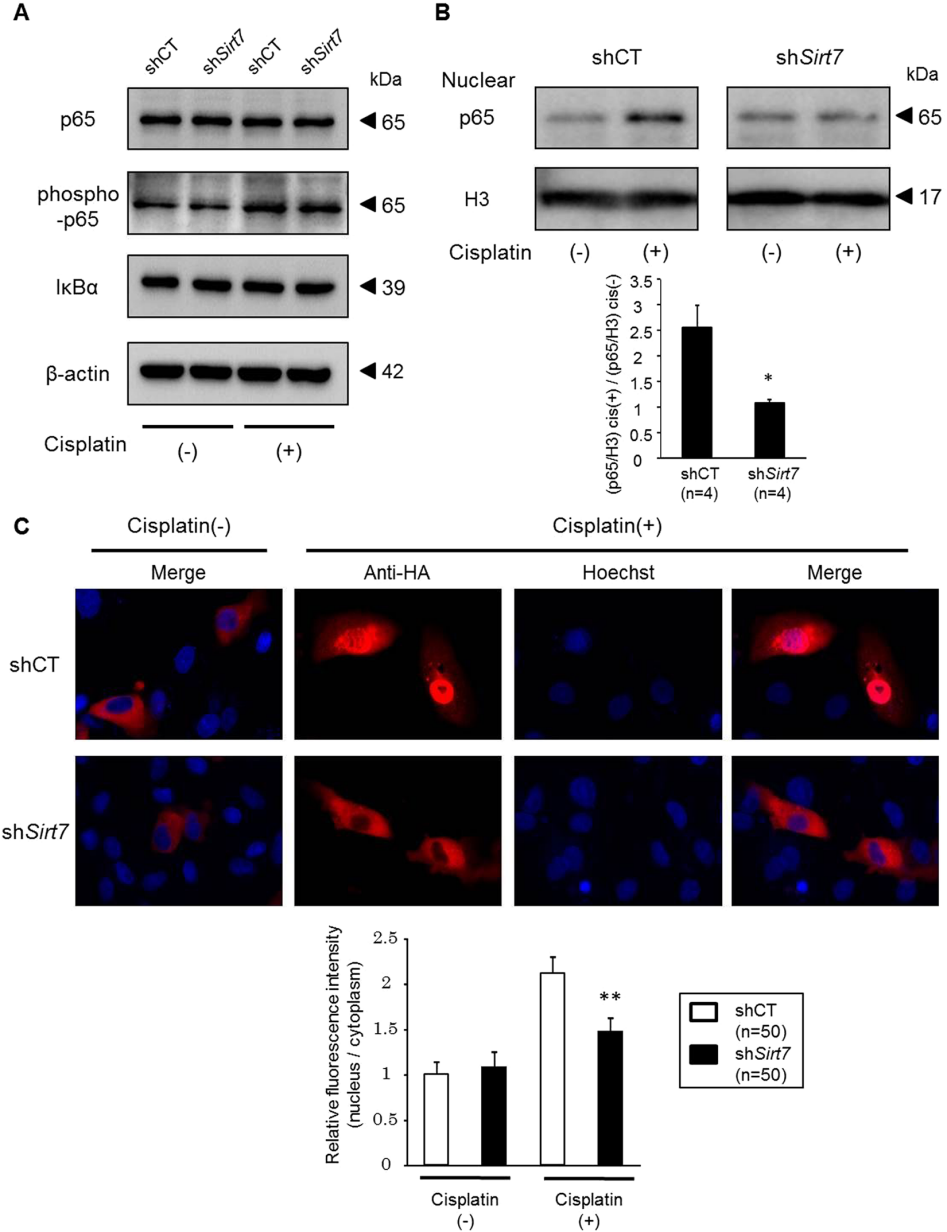
Nuclear accumulation of p65 is suppressed in *Sirt7*-deficient cells. (**A**) Protein expression of p65, phospho-p65, and IκBα in whole cell lysates of shRNA-introduced NRK-52E cells with or without 1-h cisplatin (30 μM) treatment was evaluated by western blot analysis. β-actin was used as a loading control. (**B**) Protein expression of p65 in the nuclear fraction with or without 1-h cisplatin (30 μM) treatment. Relative nuclear p65 expression was normalized by H3 (p65/H3, n = 4/group). To indicate the fold change of nuclear p65 expression by cisplatin, the value of p65/H3 with cisplatin exposure (p65/H3 cis (+)) was divided by that without cisplatin (p65/H3) cis (−)) (n = 4/group). Full-length blots are presented in Supplementary Figure S7. (**C**) Intracellular localization of p65. NRK-52E cells with or without 1-h cisplatin (30 μM) treatment were transfected with the pCI-HA-p65 plasmid, and p65 expression was evaluated by HA immunostaining. For nuclear staining, Hoechst 33342 was used. Relative fluorescence intensity is represented by the ratio of nuclear fluorescence intensity in the ROI to cytoplasmic fluorescence intensity in the ROI as measured by ImageJ software (n = 50/group). *p < 0.05, **p < 0.01. Data are expressed as the mean ± SEM.

Western blot analysis revealed that the expression levels of p65, phospho-p65, and IκBα proteins in whole cell lysates were unchanged between the control and *Sirt7* KD NRK-52E cells (Fig. 7A). We next investigated nuclear p65 expression after cisplatin treatment. Exposure of the control NRK-52E cells to cisplatin markedly increased the levels of nuclear p65, whereas this increase was significantly blunted in the *Sirt7* KD NRK-52E cells (Fig. 7B). The suppression of increased nuclear p65 expression by cisplatin was also detected in *Sirt7* KO mouse embryonic fibroblast cells (Supplementary Fig. S7). We next examined the effect of SIRT7 on the cellular location of p65 by immunofluorescence microscopy. Transiently transfected p65 was mainly cytoplasmic in both untreated control and *Sirt7* KD NRK-52E cells (Fig. 7C). After cisplatin stimulation, p65 staining was detected in the nucleus of the control cells, but p65 signals mostly remained cytoplasmic after cisplatin exposure in the *Sirt7* KD cells (Fig. 7C). Relative nuclear p65 fluorescence intensity was significantly decreased in *Sirt7* KD NRK-52E cells.

**Figure 8:**
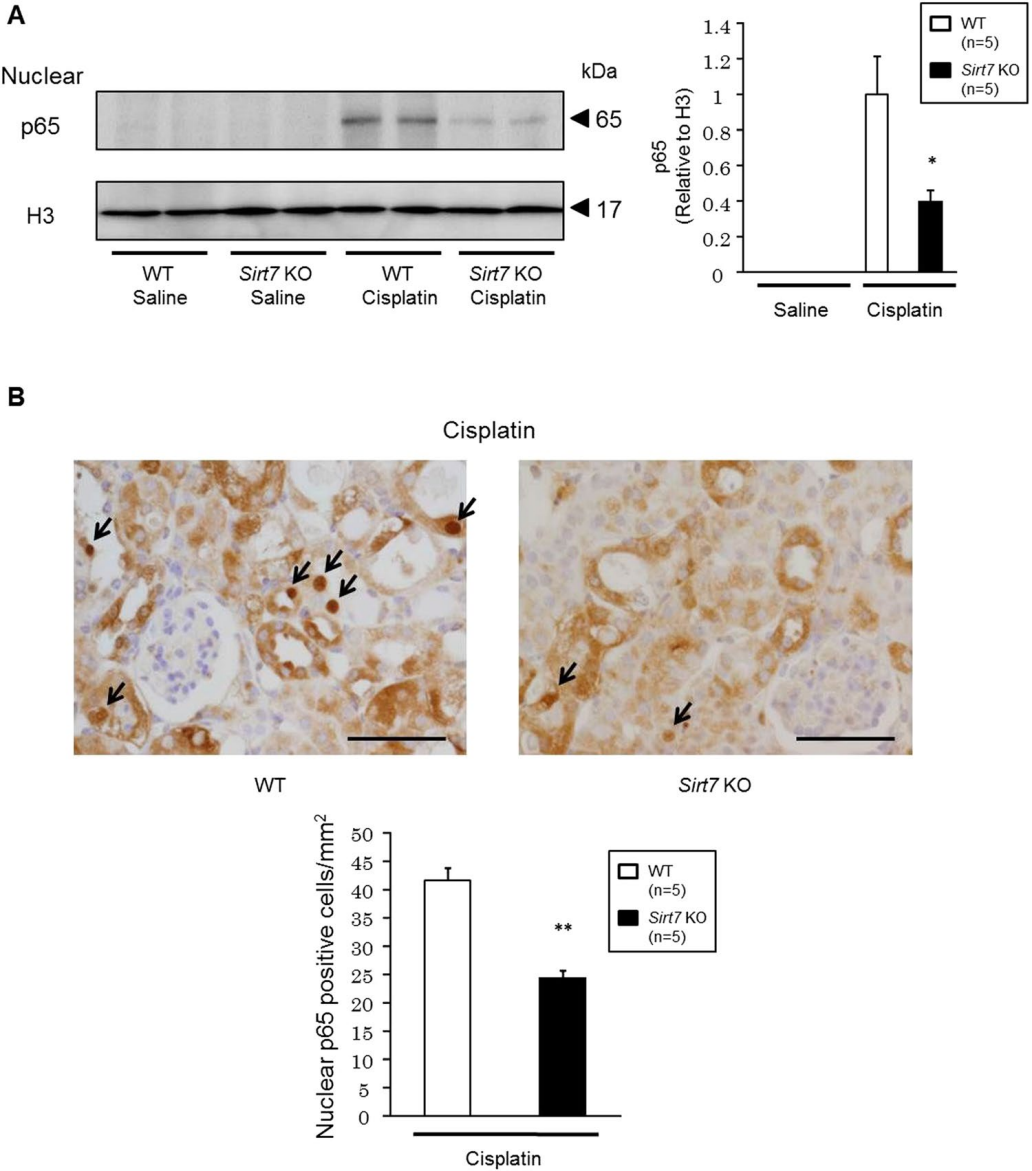
Nuclear accumulation of p65 is suppressed in the kidney of cisplatin-treated *Sirt7* KO mice. (**A**) Protein expression of p65 in the nuclear fraction of mouse kidney was evaluated by western blot analysis. H3 was used as a loading control (n = 5/group) (**B**) Nuclear p65 protein expression in tubular cells was evaluated by p65 immunostaining (n = 5/group). The arrows indicate p65-expressing nuclei. Scale bars: 50 µm. *p < 0.05, **p < 0.01. Data are expressed as the mean ± SEM.

Finally, we examined nuclear p65 protein expression in the kidney of *Sirt7* KO mice. Western blotting analysis revealed that nuclear p65 protein expression was significantly decreased in the kidney of *Sirt7* KO mice compared with that in WT mice after cisplatin administration (Fig. 8A). Immunohistochemical analysis also showed that the number of nuclear p65-positive tubular cells was significantly decreased in the *Sirt7* KO mice compared with that in the WT mice (Fig. 8B). These results suggest that the loss of SIRT7 suppresses the nuclear accumulation of p65 after cisplatin exposure both *in vitro* and *in vivo*.

## Discussion

Inflammation and ROS-generated oxidative stress are involved in cisplatin-induced nephrotoxicity^1–3^. Previous studies have indicated that SIRT1 and SIRT3 are protective in cisplatin-induced kidney injury^6–8^. Interestingly, SIRT7 apparently plays an opposite role. TNF-α plays a critical role in the pathogenesis of cisplatin-induced AKI^23,24^ and stimulates the expression of a number of inflammatory cytokines and chemokines (e.g., MIP-2α). NADPH oxidase is a significant source of ROS. TNF-α also enhances ROS production by increasing the expression of NOX2 and p47phox, which are subunits of the NADPH oxidase complex^42,43^. The expression of *Tnfa*, *Cxcl2* (encoding MIP-2α), *Cybb* (encoding NOX2), and *Ncf1* (encoding p47phox) mRNA was significantly reduced in the cisplatin-treated kidney of *Sirt7* KO mice. Thus, SIRT7 deficiency might ameliorate cisplatin-induced renal injury at least in part by reducing the expression of TNF-α. SIRT2 deficiency ameliorates lipopolysaccharide-induced acute tubular injury and suppresses renal failure with decreased renal *Cxcl2* mRNA expression^44^. Thus, the reduced expression of *Cxcl2* might also be involved in the amelioration of cisplatin-induced kidney injury.

Inflammation is one of the key processes that contributes to renal injury at an acute stage in the UUO model^22^. The expression levels of *Tnfa* and *Cxcl2* were significantly decreased in the obstructed kidney of *Sirt7* KO mice. Decreased expression of inflammation-related genes is also detected in the heart of *Sirt7* KO mice after myocardial infarction and in the white adipose tissue of high-fat diet-fed *Sirt7* KO mice^10,14^. These findings strongly suggest that SIRT7 plays critical roles in the regulation of inflammation. In the present study, we identified that NF-κB activity and the expression of TNF-α are decreased in cisplatin-treated *Sirt7* KD NRK-52E kidney cells. However, considering the important roles of macrophages in the progression of inflammation and the anti-inflammatory roles of SIRTs in macrophages^45,46^, further investigation is required to clarify the relative contribution of TNF-α production by SIRT7 in infiltrating immune cells using macrophage-specific *Sirt7* KO mice.

SIRTs regulate NF-κB function in multiple ways. SIRT6 suppresses NF-κB target gene expression by deacetylating histone H3 lysine 9 and destabilizing the binding of NF-κB to chromatin^34^. Phosphorylation of p65 stimulates acetylation at Lys 310, and this acetylation enhances the transcriptional activity of NF-κB^47^. SIRT1 and SIRT2 inhibit NF-κB activity through deacetylation of p65 at Lys 310^32,33^. We found that total p65 expression levels were unchanged, but nuclear p65 expression after cisplatin exposure was decreased in *Sirt7* KD NRK-52E cells. We also found that *Sirt7* deficiency in mouse aortic smooth muscle cells led to the suppression of lipopolysaccharide-induced p65 nuclear accumulation (unpublished data). Thus, SIRT7 may regulate the nuclear accumulation of NF-κB p65. SIRT7 is located in the nucleus, while p65 is predominantly present in the cytosol of unstimulated cells. However, there is substantial evidence indicating that NF-κB-IκBα complexes are not static in their localization but are shuttled continuously between the nucleus and cytoplasm^48–50^. It may not be unanticipated, therefore, that SIRT7 binds to p65 in the nucleus and regulates this shuttling. Although SIRT7 could not deacetylate p65, recent studies have shown that SIRT7 exhibits desuccinylase or defatty-acylase activity^51,52^. Therefore, SIRT7 might regulate nuclear p65 translocation by regulating non-acetyl-lysine modifications. Lysine acetylation affects protein-protein interactions^53^. Alternatively, SIRT7 might regulate p65 nuclear translocation indirectly by deacetylating p65-interacting proteins. Further studies are necessary to address how SIRT7 regulates p65 translocation to the nucleus.

Several studies have shown that KD of *Sirt7* increases apoptosis *in vitro*^54,55^. We also found that *Sirt7* KD promoted apoptosis in NRK-52E cells (data not shown). In contrast, fewer TUNEL-positive cells were noted in the kidney of *Sirt7* KO mice compared with WT mice. Cisplatin-induced proinflammatory cytokines increase apoptosis in renal cells^56,57^. Thus, inhibition of the recruitment and accumulation of inflammatory cells by the suppression of cytokines and chemokines in *Sirt7* KO mice might be responsible for the overall anti-apoptosis effect *in vivo*. Further studies are also needed to clarify the relationship between SIRT7 and apoptosis.

In conclusion, these results demonstrated that SIRT7 deficiency ameliorated cisplatin-induced AKI in mice. Our findings suggest that SIRT7 is an important novel therapeutic target for cisplatin-induced AKI.

## Methods

### Animal Experiments

All experimental procedures were performed in accordance with the guidelines of the Institutional Animal Committee of Kumamoto University and were approved by the Committee on Animal Research at Kumamoto University. Except when indicated, 10-week-old WT and *Sirt7* KO mice with a C57/BL6J background were used for this study. The generation of *Sirt7* KO mice and *Sirt7* FRT/floxed mice was described previously^10,58^. The mice were housed in a temperature- and humidity-controlled environment with a 12:12-h light–dark cycle and were fed a standard normal diet *ad libitum* with free access to water. Cisplatin (cis-diammineplatinum(II) dichloride; Sigma Aldrich, St. Louis, MO) was dissolved in normal saline at a concentration of 1.0 mg/mL, and was given by intraperitoneal injection of 20 mg/kg body weight as described previously^59^. Metabolic cages were used for 24-h urine collections. Blood and kidneys were harvested at 72 h after cisplatin injection. Serum chemistry analysis was performed by a commercial laboratory (SRL, Tokyo, Japan).

### Urinary NGAL Concentrations

We collected 24-h urine samples at days 1, 2, and 3 after cisplatin injection. Urinary NGAL concentrations were measured using a mouse NGAL ELISA Kit (BioPorto Diagnostics, Gentofte, Denmark). For creatinine adjustment, urine creatinine levels were measured using a Lab Assay Creatinine Kit (Wako, Osaka, Japan).

### Histology and Tubular Injury Score

The kidneys were fixed with 10% formalin neutral buffer solution and embedded in paraffin. Sections (4-μm thick) were stained using Periodic acid-Schiff and examined under a light microscope. The tubular injury score was calculated on a scale of 0–5 on the basis of the percentage of tubules with necrosis, cast formation, dilation, or loss of the brush border: 0, 0%; 1, 1–10%; 2, 11–25%; 3, 26–45%; 4, 46–75%; and 5, 76–100%^60^. A pathologist evaluated 5 randomly selected fields per section of the mouse kidney at a magnification of ×400 in a blind manner.

### β-Galactosidase (lacZ) Staining

Kidney sections harvested from *Sirt7* FRT/floxed and WT mice were fixed with 4% paraformaldehyde for 4 h at 4 °C. Fixed sections were washed with 0.02% NP-40 in phosphate-buffered saline (PBS) for 20 min and PBS for 20 min 3 times. After washing, the sections were transferred to an X-gal solution (0.5 mg/mL X-gal, 5 mM K_3_Fe(CN)_6_, 5 mM K_4_Fe(CN)_6_, 1 mM MgCl_2_) and incubated at 37 °C overnight.

### Immunohistochemical Staining

Deparaffinized sections were subjected to microwave pretreatment with a pH 6.0 citrate buffer for p65 immunostaining or pretreatment with proteinase K for F4/80 immunostaining^61^. After the reaction of each primary antibody (anti-p65 antibody, #8242, Cell Signaling Technology, Beverly, MA, and anti-F4/80 antibody clone CI:A3-1, Serotec, Oxford, UK), the samples were incubated with horseradish peroxidase (HRP)-labeled goat anti-rat or goat anti-rabbit antibodies (Nichirei, Tokyo, Japan). The reaction was visualized using the diaminobenzidine system (Nichirei).

For immunostaining of SIRT7 and lectin, deparaffinized sections were incubated in HistoVT One solution (Nacalai Tesque, Kyoto, Japan) for 40 min at 90 °C. After reaction with an anti-SIRT7 antibody (1:300, #5360, Cell Signaling Technology), the sections were reacted with a biotinylated goat anti-rabbit IgG antibody and HRP-labeled streptavidin (Nichirei). The reaction was visualized with diaminobenzidine (brown) as above. After the sections were rinsed with 100 mM glycine-HCL buffer (pH 2.2) to remove the primary and secondary antibodies, the sections were reacted with biotinylated PHA-E (Vector Laboratories, Burlingame, CA) or DBA (Vector Laboratories) and then incubated with HRP-labeled streptavidin. The reaction was visualized with HistoGreen (Linaris, Heidelberg, Germany) (green).

### TUNEL Assay

TUNEL assay was performed with a commercially available detection kit (*In situ* Apoptosis Detection Kit; Takara, Shiga, Japan).

### Cell Culture

Rat kidney proximal tubular cells (NRK-52E) were obtained from ATCC (Manassas, VA). NRK-52E cells were maintained in Dulbecco’s modified Eagle’s medium containing 25 mM glucose, 1.0 mM pyruvate, 5% (v/v) fetal bovine serum, and 0.1% (v/v) penicillin/streptomycin at 37 °C in 5% CO_2_.

### SIRT7 Gene Silencing

For KD of SIRT7, oligonucleotides encoding SIRT7 shRNA (target sequence: sh*Sirt7*, 5′-GGGACACCATTGTGCACTT-3′) were cloned into the pSIREN-RetroQ expression vector (Clontech, Mountain View, CA). After the pSIREN-RetroQ-SIRT7 or pSIREN-RetroQ-control vector was transfected into Plat-E cells, the retrovirus-containing medium was mixed with Polybrene and added to NRK-52E cells for infection. Selection was achieved using puromycin treatment (5 µg/mL).

### Quantitative Real-time PCR

Total RNA from mouse kidneys and NRK-52E cells was extracted using the TRIzol reagent (Invitrogen, Carlsbad, CA) and an RNeasy Mini Kit (QIAGEN, Hilden, Germany). cDNA synthesis was then achieved with a Prime Script RT Reagent Kit (Takara). Quantitative real-time PCR (qPCR) was performed using TaqMan probes for mouse *Tnfa*, *Il1b*, *Il6* (Sigma Aldrich), *Ccl2*, *Cxcl2*, *Cybb*, *Ncf1*, *Sod1*, *Sod2*, *Slc22a2*, *Slc47a1*, and *Gapdh*, and rat *Tnfa*, *Il1b*, *Il6, Ccl2*, *Cxcl2*, *Cybb*, *Ncf1*, and *Gapdh* (Applied Biosystems, Foster City, CA) in the Light Cycler 480 Sequence Detector System (Roche Diagnostics, Mannheim, Germany), or primers for mouse *Sirt1–7, Acox1*, *Cycs*, *Cat*, and *Gapdh* with SYBR Premix Ex Taq II (Takara) in an ABI 7300 Thermal Cycler (Applied Biosystems). The results were analyzed statistically based on ΔCT values (Ct _gene of interest_ − CT _GAPDH_). Relative gene expression was obtained using the ΔΔCt method (Ct _sample_ − Ct _calibrator_).

### Western Blotting

Total lysates of cells and kidney tissues were obtained by lysis in RIPA Buffer (50 mM Tris-HCl [pH 8.0], 150 mM NaCl, 0.1% sodium dodecyl sulfate (SDS), 1% NP-40, 5 mM EDTA, 0.5% sodium deoxycholate, 20 mg/mL Na3VO4, 10 mM NaF, and 1 mM PMSF) with a protease inhibitor cocktail (Nacalai Tesque). Nuclear fractions of cells and kidney tissues were prepared using a LysoPure™ Nuclear and Cytoplasmic Extractor Kit (Wako) according to the manufacturer’s protocol. Aliquots of proteins were subjected to sodium dodecyl sulfate-polyacrylamide gel electrophoresis in a reduced condition and transferred to a polyvinylidene fluoride membrane (Immobilon-P; Millipore, Bedford, MA), which was probed with the primary antibodies. After incubation with the secondary antibodies, proteins were visualized using Chemi-Lumi One Super (Nacalai Tesque) and a LAS-1000 imaging system (Fujifilm, Tokyo, Japan). Primary antibodies included an anti-SIRT7 antibody (#5360, Cell Signaling Technology), anti-cleaved caspase 3 antibody (#9661, Cell Signaling Technology), anti-p65 antibody (#4764, Cell Signaling Technology), anti-phospho-p65 antibody (#3031, Cell Signaling Technology), anti-IκBα antibody (#9242, Cell Signaling Technology), anti-β-actin antibody (A5060, Sigma Aldrich), anti-GAPDH antibody (#2118, Cell Signaling Technology), and anti-histone H3 antibody (#39163, Active Motif).

### Immunofluorescence

Each chamber of a 4-chamber 35-mm glass bottom dish were seeded with 5.0 × 10^3^ shRNA-introduced NRK-52E cells and incubated for 24 h. Then, the cells were transfected with 0.5 μg pCI-HA-p65 expression plasmid in each chamber using jetPRIME (Polyplus-transfection, Strasbourg, France). At 24 h after transfection, the cells were treated with or without 30 μM cisplatin for 1 h. Then, the cells were washed with PBS and fixed in a 10% formalin neutral buffer solution for 15 min. The fixed cells were incubated with 0.1% Triton X-100 in PBS for 10 min to permeabilize the cells. In the blocking step, the cells were covered with Blocking One (Nacalai Tesque) for 20 min. The cells were incubated at 4 °C overnight with primary antibodies against the hemagglutinin (HA) tag (1:500, Roche Diagnostics). To detect the primary antibody, the sections were incubated with anti-rat Alexa Fluor 555-conjugated antibodies (1:1000, Life Technologies). Hoechst 33342 (Dojindo, Kumamoto, Japan) was used for nuclear staining. The fluorescence intensity in the regions of interests (ROI) in the nucleus and cytoplasm was measured using ImageJ software, as described previously^62^. Relative fluorescence intensity is represented by the ratio of nuclear fluorescence intensity to cytoplasmic fluorescence intensity.

### Luciferase Assay

The NF-κB luciferase reporter plasmid (pNF-κB-Luc, Takara) was used for measuring NF-κB activity, and the pRL-TK plasmid was used as an internal control. The pCI-HA-p65 expression plasmid was used for examining the expression of p65, and the pCI-HA-control plasmid was used as a control. The pCI-HA-p65 expression plasmid was constructed as follows: the HA tag was inserted into the pCI-p65 expression plasmid (gifted from Dr. Takashi Minami) by PCR using HA-containing primers. The pcDNA3-FLAG-*Sirt7* expression plasmid was used for the expression of *Sirt7*, and the pcDNA3-FLAG-control plasmid was used as a control.

The shRNA-introduced NRK-52E cells were seeded in 12-well plates at 7.5 × 10^4^ cells/well and maintained for 24 h. Subsequently, the pNF-κB-Luc plasmid and pRL-TK plasmid were co-transfected into the cells with expression plasmids using jetPRIME (Polyplus-transfection). The luciferase assay was performed 24 h later with the Dual-Luciferase reporter assay system (Promega, Madison, WI).

### Immunoprecipitation

HEK293T cells were co-transfected with pcDNA3-FLAG-p65 and either pcDNA3-HA, pcDNA3-HA-*Sirt1*, or pcDNA3-HA-*Sirt7* using jetPRIME (Polyplus-transfection). After 24 h, the cells were lysed using a 27 G syringe in immunoprecipitation (IP) buffer (20 mM Tris-HCl [pH 7.4], 200 mM NaCl, 2.5 mM MgCl_2_, 0.5% NP-40, 1 mM PMSF, protease inhibitor cocktail [Nacalai Tesque]). The soluble cellular fractions were recovered by centrifugation at 15,000 rpm for 15 min at 4 °C. Then, 700 μg cell lysate was mixed with FLAG-tag antibody beads (Wako) and stirred at 4 °C for 15 h. After washing with IP buffer, proteins were eluted with 1 × SDS sample buffer (100 mM Tris-HCl [pH 6.8], 4% SDS, 20% glycerol, and 0.2% bromophenol blue) for western blotting. Binding of SIRT7 protein to p65 was detected with a rat anti-HA high affinity antibody (3F10; Roche Diagnostics).

### Detection of Lysine Acetylation

HEK293T cells were co-transfected with pcDNA3-FLAG-p65, pCMVβ-*p300*-myc, and either pcDNA3-HA, pcDNA3-HA-*Sirt7*, or pcDNA3-HA-*Sirt1* using jetPRIME (Polyplus-transfection). After 36 h, the cells were lysed with a 27 G syringe in IP buffer. The soluble cell fractions were recovered by centrifugation at 15,000 rpm for 15 min at 4 °C. Then, 500 μg cell lysate was mixed with FLAG-tag antibody beads (Wako) and stirred at 4 °C for 6 h. After washing with IP buffer, proteins were eluted with 1× SDS sample buffer and subjected to western blotting. Acetylation of p65 protein was detected with a Pan anti-acetyl lysine rabbit polyclonal antibody (TM-105; PTM Biolabs, Chicago, IL). pCMVβ-*p300*-myc was a gift from Tso-Pang Yao (Addgene plasmid # 30489).

### Statistical Analysis

Comparisons between subject groups were analyzed with Student’s *t*-test or the Mann–Whitney U-test. Survival rates were analyzed by the log-rank test. For comparisons among more than two groups, analysis of variance with Bonferroni’s correction was used. P-values < 0.05 were considered significant. Results are presented as the mean ± standard error of the mean and were prepared in Excel or GraphPad Prism.

### Data Availability Statement

The data generated or analyzed during this study are included in this published article (and its Supplementary Information files) or are available from the corresponding author on reasonable request.

## Electronic supplementary material

Supplementary Information
