# Supplementary material for: Sirtuin 7 Deficiency Ameliorates Cisplatin-induced Acute Kidney Injury Through Regulation of the Inflammatory Response
Source: Sci Rep. 2018 Apr 12;8:5927. doi: 10.1038/s41598-018-24257-7 (PMC5897539; doi:10.1038/s41598-018-24257-7)
Supplement: Supplementary file 1 — Supplementary Information [file 41598_2018_24257_MOESM1_ESM.pdf]

Sirtuin 7 Deficiency Ameliorates Cisplatin-induced Acute Kidney Injury Through Regulation of the Inflammatory Response

Yoshikazu Miyasato[1,2], Tatsuya Yoshizawa[1,5], Yoshifumi Sato[1,5], Terumasa Nakagawa[2,5], Yuko Miyasato[3,5], Yutaka Kakizoe[2], Takashige Kuwabara[2], Masataka Adachi[2], Alessandro Ianni[4], Thomas Braun[4], Yoshihiro Komohara[3], Masashi Mukoyama[2], and Kazuya Yamagata[1,*]

[1]Department of Medical Biochemistry, Faculty of Life Sciences, Kumamoto University, 1-1-1 Honjo, Chuo-ku, Kumamoto, Japan

[2]Department of Nephrology, Faculty of Life Sciences, Kumamoto University, 1-1-1 Honjo, Chuo-ku, Kumamoto, Japan

[3]Department of Cell Pathology, Faculty of Life Sciences, Kumamoto University, 1-1-1 Honjo, Chuo-ku, Kumamoto, Japan

[4]Department of Cardiac Development and Remodeling, Max Planck Institute for Heart and Lung Research, Bad Nauheim, Germany

[5]These authors contributed equally to this work

Address correspondence to: Kazuya Yamagata, MD, PhD
Department of Medical Biochemistry, Faculty of Life Sciences, Kumamoto University, 1-1-1 Honjo, Chuo-ku, Kumamoto 860-8556, Japan
Phone: +81-96-373-5070; Fax: +81-96-364-6940
E-Mail: k-yamaga@kumamoto-u.ac.jp

Figure S1

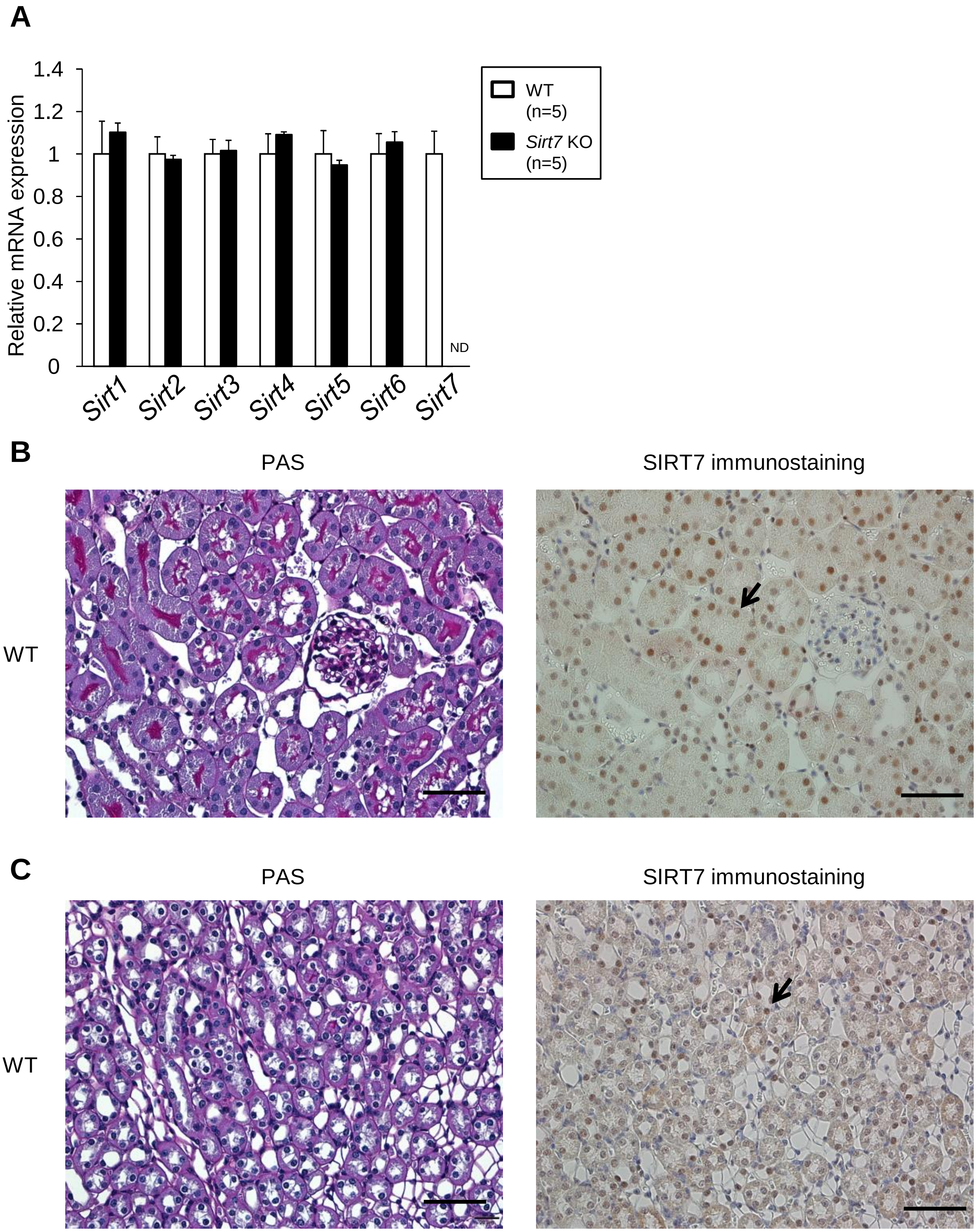

A
1.4
1.2
1
0.8
0.6
0.4
0.2
0
Relative mRNA expression
WT
(n=5)
Sirt7 KO
(n=5)
ND
Sirt1
Sirt2
Sirt3
Sirt4
Sirt5
Sirt6
Sirt7
B
PAS
SIRT7 immunostaining
WT
C
PAS
SIRT7 immunostaining
WT

Figure S2

Cisplatin

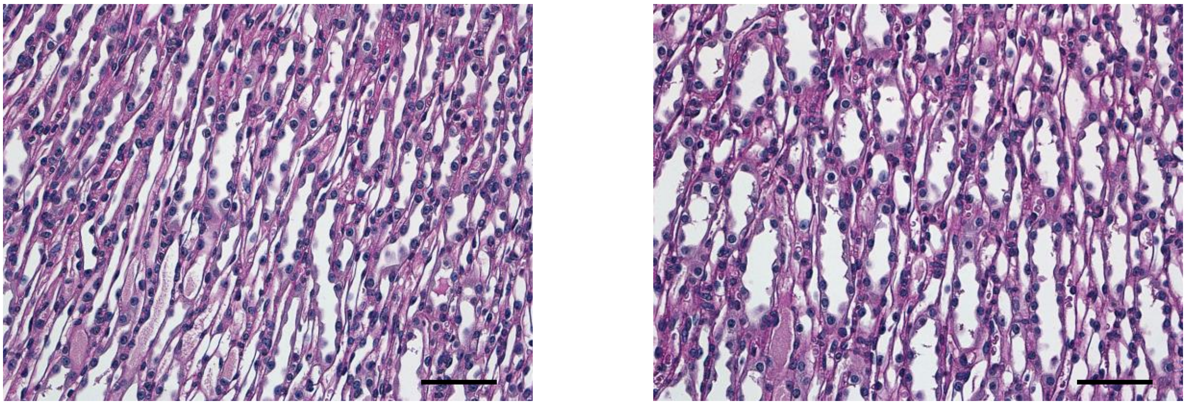

WT

Sirt7 KO

Figure S3

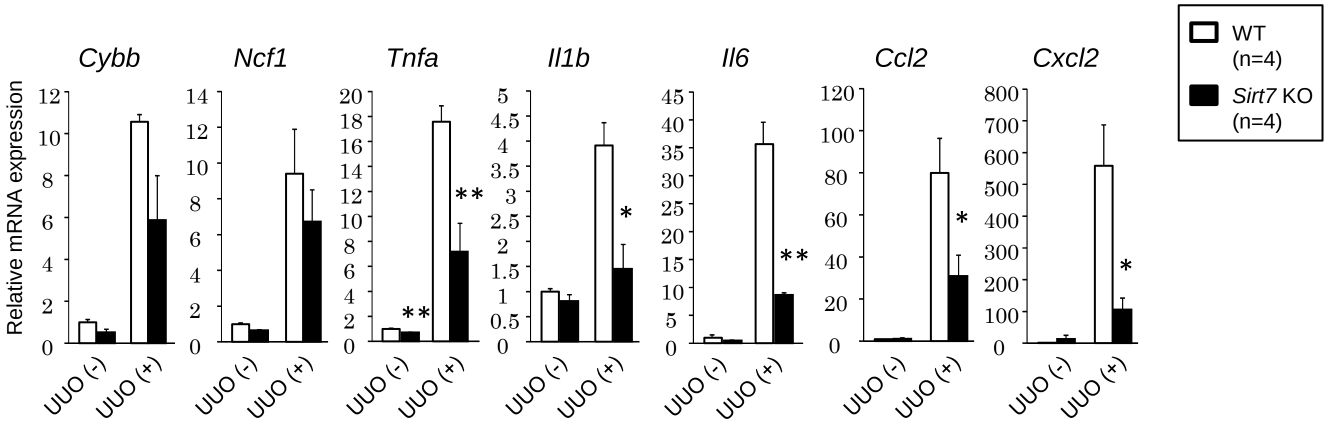

Cybb
Ncf1
Tnfa
Il1b
Il6
Ccl2
Cxcl2
Relative mRNA expression
UUO (-)
UUO (+)
**
*
WT (n=4)
Sirt7 KO (n=4)

Figure S4

Cisplatin

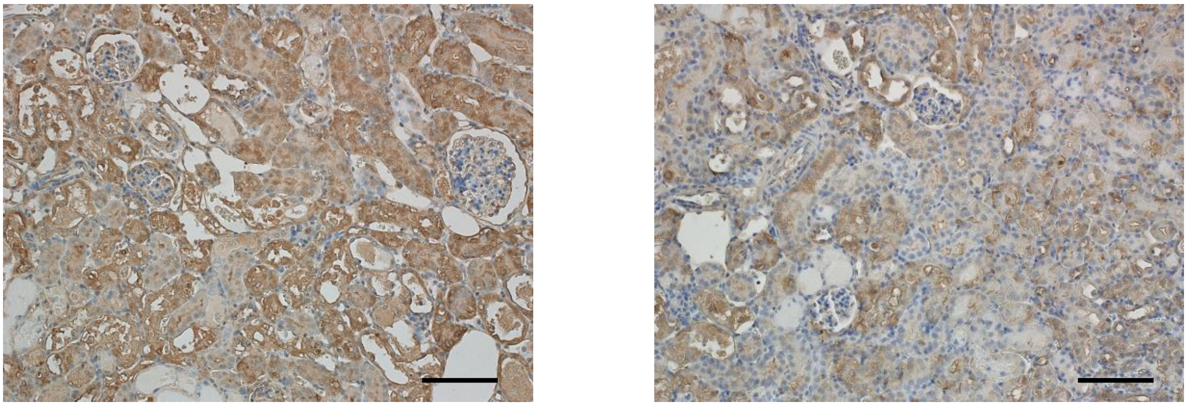

WT

Sirt7 KO

Figure S5

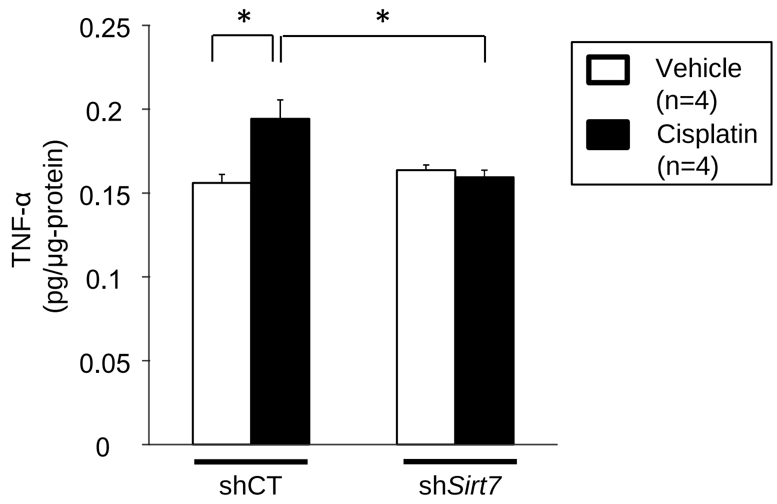

*
*
0.25
0.2
0.15
0.1
0.05
0
TNF-α
(pg/μg-protein)
shCT
sh*Sirt7*
Vehicle
(n=4)
Cisplatin
(n=4)

Figure S6

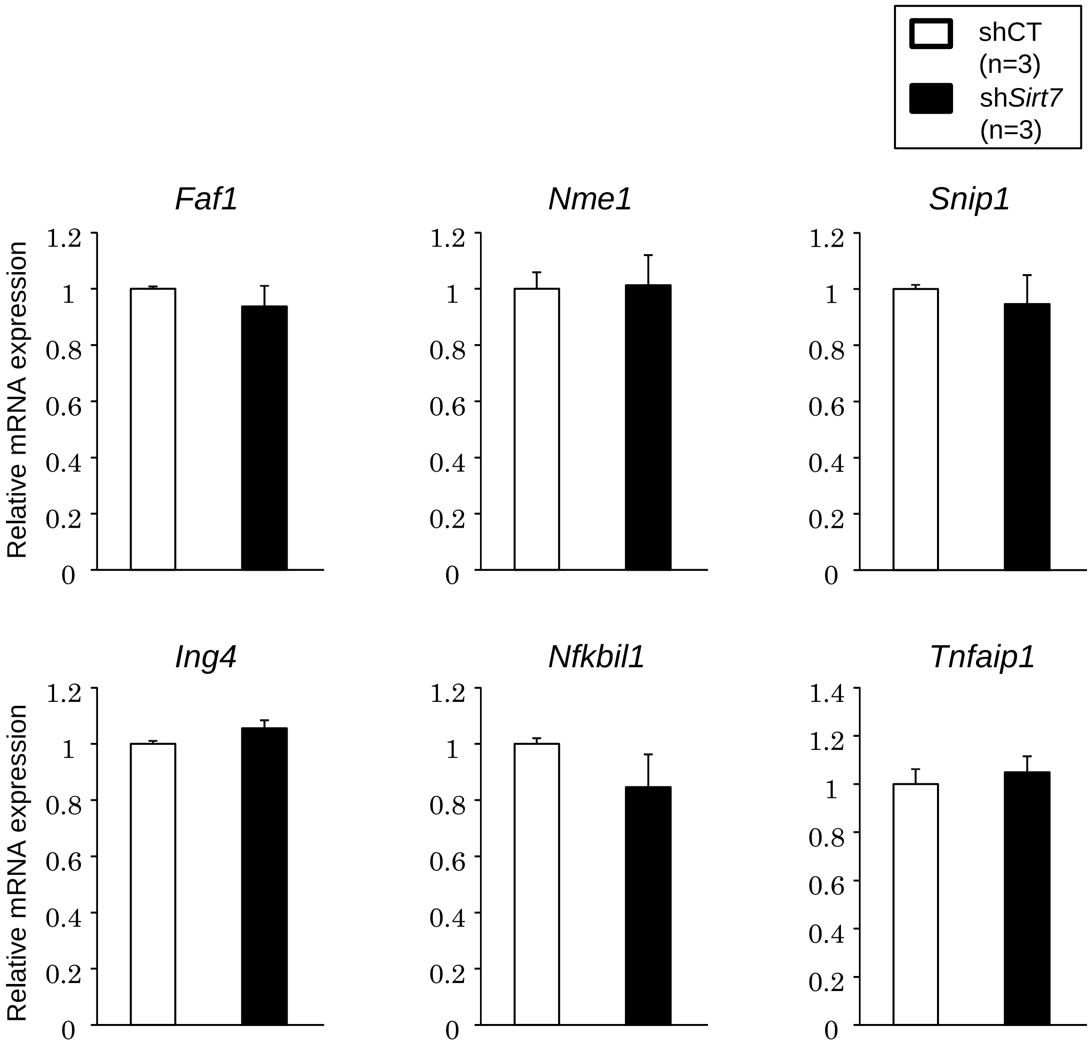

shCT
(n=3)
shSirt7
(n=3)
Faf1
Nme1
Snip1
Ing4
Nfkbil1
Tnfaip1
Relative mRNA expression
Relative mRNA expression
1.4
1.2
1
0.8
0.6
0.4
0.2
0

Figure S7

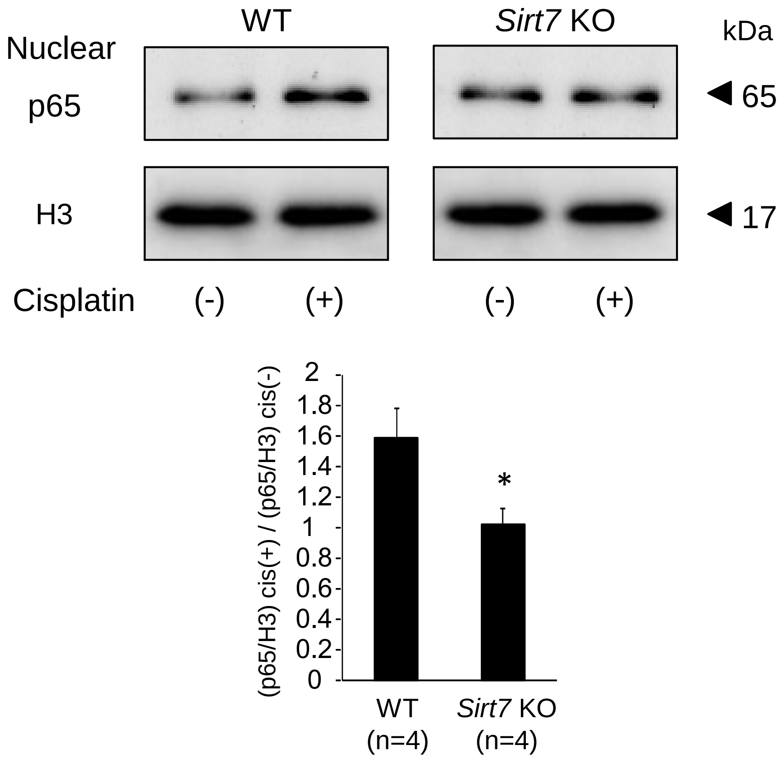
WT
Sirt7 KO
kDa
Nuclear
p65
65
H3
17
Cisplatin
(-)
(+)
(-)
(+)
(p65/H3) cis(+) / (p65/H3) cis(-)
2
1.8
1.6
1.4
1.2
1
0.8
0.6
0.4
0.2
0
*
WT
(n=4)
Sirt7 KO
(n=4)

Figure S8

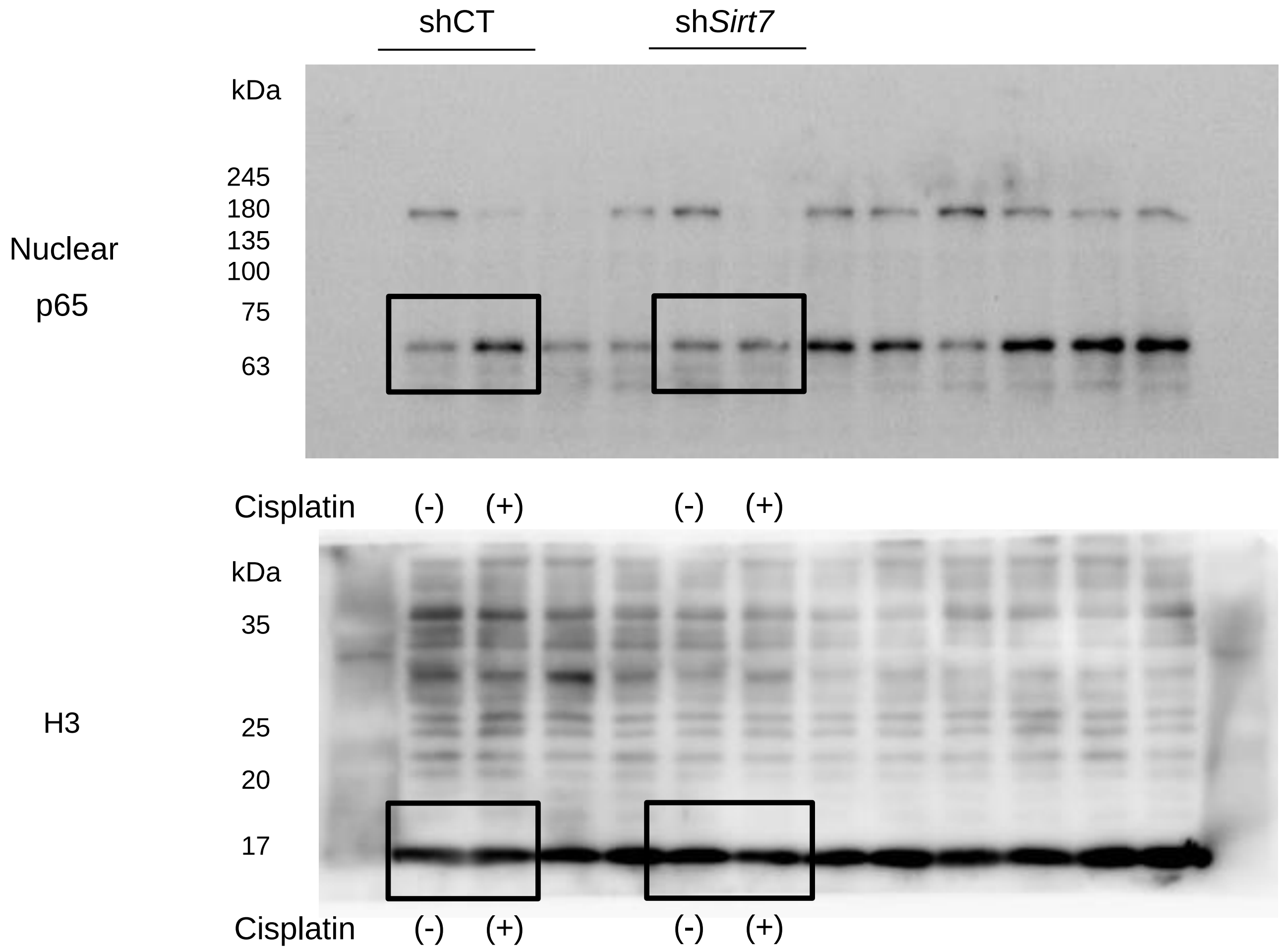
shCT
shSirt7
kDa
245
180
135
100
75
63
Nuclear
p65
Cisplatin
(-)
(+)
(-)
(+)
kDa
35
25
20
17
H3
Cisplatin
(-)
(+)
(-)
(+)

Figure S9

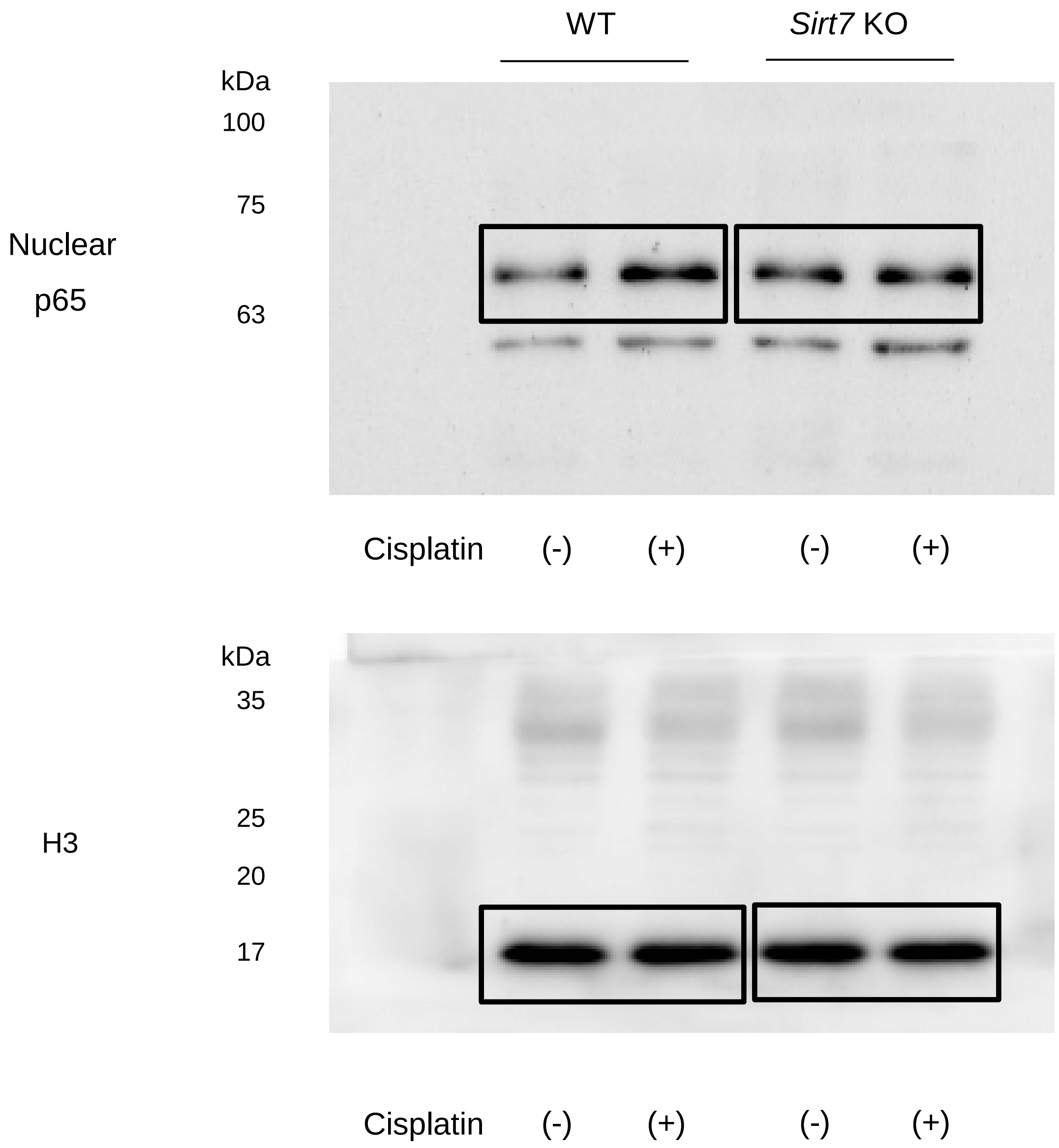
WT
Sirt7 KO
kDa
100
75
63
Nuclear
p65
Cisplatin
(-)
(+)
(-)
(+)
kDa
35
25
20
17
H3
Cisplatin
(-)
(+)
(-)
(+)

Supplementary Figure Legends

Supplementary Figure S1

(A) mRNA expression of *Sirt1-7* in the mice kidneys was determined by real-time PCR (n = 5/group). The abundance of each mRNA type was normalized using GAPDH. ND, not detected. (B) Representative photomicrographs (×400) of PAS staining and immunohistochemical staining for SIRT7 in the cortical region of WT mouse kidney. SIRT7 expression in proximal tubular cells is represented. (C) Representative photomicrographs (×400) of PAS staining and immunohistochemical staining for SIRT7 in the inner medullary region of WT mouse kidney. SIRT7 expression in collecting duct cells is represented. The arrows indicate SIRT7-expressing nuclei. Scale bars: 50 µm.

Supplementary Figure S2

Representative photomicrographs (×400) of the inner medullary region of the kidney. Kidney sections were stained with PAS. Scale bars: 50 µm.

Supplementary Figure S3

Oxidative stress-related and inflammation-related mRNA expression in the kidney of UUO model mice was determined by real-time PCR. This experiment utilized 12-week-old male mice (n = 4/group). The abundance of each mRNA type was normalized using GAPDH. *p < 0.05, **p < 0.01. Data are expressed as the mean ± SEM.

Supplementary Figure S4

Representative photomicrographs (×200) of immunohistochemical staining for MCP1 in the cisplatin-treated kidney of WT and *Sirt7* KO mice. Scale bars: 100 µm.

Supplementary Figure S5

Protein expression of TNF-α in shRNA-introduced NRK-52E cells was determined using rat TNF-α ELISA kits. TNF-α protein amount was adjusted by the protein amount of total cell lysate (n = 4/group). The cells were exposed with or without 30 µM cisplatin for 24 h. *p < 0.05, **p < 0.01. Data are expressed as the mean ± SEM.

Supplementary Figure S6

mRNA expression of the genes involved in the NF-κB signaling pathway was determined by real-time PCR. The abundance of each mRNA type was normalized using GAPDH (n = 3/group). *$p < 0.05$, **$p < 0.01$. Data are expressed as the mean ± SEM.

Supplementary Figure S7

Protein expression of p65 in the nuclear fraction in whole cell lysate of MEF cells with or without 1-h cisplatin (30 μM) exposure (n = 4/group). Full-length blots are presented in Supplementary Figure S9.

Supplementary Figure S8

Full-length blots of Figure 7B are presented.

Supplementary Figure S9

Full-length blots of Supplementary Figure S7 are presented.

Supplementary Methods

Immunohistochemical Staining

Deparaffinized sections were subjected to microwave pretreatment with a pH 6.0 citrate buffer. After reaction with the primary antibody (anti-MCP1 antibody, ab25124; Abcam, Cambridge, UK), the samples were incubated with an HRP-labeled goat anti-rabbit antibody (Nichirei). The reaction was visualized using the diaminobenzidine system (Nichirei).

Unilateral Ureteral Obstruction (UUO) Model

Twelve-week-old WT and *Sirt7* KO mice with a C57/BL6J background were used for preparing the UUO model mice. UUO was performed as described previously.[1] The kidneys were harvested at 3 days after surgery.

Quantitative Real-time PCR

Quantitative real-time PCR was performed using TaqMan probes for mouse *Tnfa*, *Il1b*, *Il6* (Sigma Aldrich), *Ccl2*, *Cxcl2*, *Cybb*, *Ncf1*, and *Gapdh* (Applied Biosystems) in the Light Cycler 480 Sequence Detector System (Roche

Diagnostics), or primers for mouse *Sirt1–7* and *Gapdh*, and rat *Faf1*, *Nme1*, *Snip1*, *Ing4*, *Nfkbil1, Tnfaip1*, and *Gapdh* with SYBR Premix Ex Taq II (Takara) in an ABI 7300 Thermal Cycler (Applied Biosystems). The results were analyzed statistically based on the ΔCT values ($Ct_{\text{gene of interest}} - CT_{\text{GAPDH}}$). Relative gene expression was obtained using the ΔΔCt method ($Ct_{\text{sample}} - Ct_{\text{calibrator}}$).

TNF-α ELISA

TNF-α levels in cell lysates were measured with a TNF-α Rat ELISA Kit (Invitrogen). shRNA-transfected NRK-52E cells were seeded in 12-well culture plates and grown to confluence. Subsequently, the culture medium was replaced with 30 μM cisplatin-containing culture medium. Then, 24 h later, the cells were washed using PBS and lysed with 250 μL buffer, comprising 0.5% Triton X-100 in PBS with a protease inhibitor cocktail (Nacalai Tesque). Cell lysates were homogenized by the vigorous vortex method and ultrasonication. Total protein concentration was determined using a BCA protein assay and lysates were analyzed for TNF-α.

Mouse Embryonic Fibroblast (MEF) Isolation

Dissected mouse embryos of WT and *Sirt7* KO mice (E13.5) were minced and then incubated with 0.05% trypsin-EDTA for 20 min in a 37°C incubator. After incubation, the tissues were disrupted by pipetting up and down 30 times with an equal volume of MEF medium (Dulbecco's modified Eagle's medium containing 25 mM glucose, 1.0 mM pyruvate, 10% [v/v] fetal bovine serum, and 0.1% [v/v] penicillin/streptomycin). After 5 min of centrifugation at 1500 rpm, the supernatant was removed and the pellet was resuspended in fresh MEF medium. The cell suspension was incubated for 2 min at room temperature and the supernatant was divided for cell culture.

Supplementary Reference

1. Ucero, A. C. *et al.* Unilateral ureteral obstruction: Beyond obstruction. *Int. Urol. Nephrol.* **46,** 765–776 (2014).

Supplementary Table 1

Clinical and biochemical characteristics of *Sirt7* KO mice.

WT, wild-type mouse; *Sirt7* KO, *Sirt7* knockout mouse; BW, body weight; Urine Volume, 24-h urine volume; Alb, serum albumin level; Cr, serum creatinine level; Na, serum sodium level; K, serum potassium level; Cl, serum chloride level; Ca, serum calcium level; P, serum phosphate level. Data are expressed as mean ± SEM. This experiment included 8-week-old male mice (n = 8/group).

Supplementary Table 1

	WT (n=8)			*Sirt7* KO (n=8)		
BW (g)	23.8	±	0.6	23.9	±	0.3
Urine Volume (mL)	2.4	±	0.2	2.7	±	0.3
Alb (g/dL)	2.8	±	0.05	2.9	±	0.1
Cr (mg/dL)	0.12	±	0.01	0.13	±	0.005
Na (mEq/L)	150.1	±	0.6	150.6	±	1.0
K (mEq/L)	4.3	±	0.1	4.2	±	0.1
Cl (mEq/L)	110.1	±	1.7	108.9	±	1.9
Ca (mg/dL)	8.8	±	0.1	9.0	±	0.05
P (mg/dL)	8.5	±	0.3	9.0	±	0.3
